# Survival and predictors of deaths of patients hospitalised due to COVID-19 from a retrospective and multicentre cohort study in Brazil

**DOI:** 10.1017/S0950268820002034

**Published:** 2020-09-07

**Authors:** M. M. Santos, E. E. S. Lucena, K. C. Lima, A. A. C. Brito, M. B. Bay, D. Bonfada

**Affiliations:** 1 Division of Microbiology, State University of Rio Grande do Norte, Caicó, RN, Brazil; 2 Department of Medice, Federal University of Rio Grande do Norte, Caicó, RN, Brazil; 3 Department of Public Health, Department of Dentistry, Federal University of Rio Grande do Norte, Natal, RN, Brazil; 4 Department of Health Sciences, Federal University of the Semi-Arid Region, Mossoró, RN, Brazil; 5 Department of Medicine, Infectious Diseases Department, Hospitalist Infectious Diseases, Federal University of Rio Grande do Norte, Natal, RN, Brazil

**Keywords:** Coronavirus infections, hospitalisations, risk factors, SARS virus, survival analysis

## Abstract

This study aimed to analyse the survival of patients admitted to Brazilian hospitals due to the COVID-19 and estimate prognostic factors. This is a retrospective, multicentre cohort study, based on data from 46 285 hospitalisations for COVID-19 in Brazil. Survival functions were calculated using the Kaplan–Meier's method. The log-rank test compared the survival functions for each variable and from that, hazard ratios (HRs) were calculated, and the proportional hazard model was used in Cox multiple regression. The smallest survival curves were the ones for patients at the age of 68 years or more, black/mixed race, illiterate, living in the countryside, dyspnoea, respiratory distress, influenza-like outbreak, O_2_ saturation <95%, X-ray change, length of stay in the intensive care unit (ICU), invasive ventilatory support, previous heart disease, pneumopathy, diabetes, Down's syndrome, neurological disease and kidney disease. Better survival was observed in the influenza-like outbreak and in an asthmatic patient. The multiple model for increased risk of death when they were admitted to the ICU HR 1.28, diabetes HR 1.17, neurological disease HR 1.34, kidney disease HR 1.11, heart disease HR 1.14, black or mixed race of HR 1.50, asthma HR 0.71 and pneumopathy HR 1.12. This reinforces the importance of socio-demographic and clinical factors as a prognosis for death.

## Introduction

During the early 20th century, the medical field supported an optimistic discourse on technological advances in the area and the control of infectious diseases, which for many centuries were the main causes of mortality in the world. However, several pathogens that have evolved or increased their ability to infect human beings have shown that curing communicable diseases must be a challenge for scientists for a long time to come. Examples of this are the SARS-CoV, MERS-CoV and now SARS-CoV-2 viruses, initially discovered as zoonoses which managed to change their transmission mechanism and start promoting direct human-to-human infection [1].

The infection caused by SARS-CoV-2 was classified as a differentiated clinical condition, which led to its inclusion as a new one, called Coronavirus Disease 2019 (COVID-19). The first cases were reported in the peninsula of Wuhan in China, in December 2019, characterised by the high transmissibility power and causing of a severe acute respiratory dysfunction, with variations between mild, moderate and severe forms, a mortality rate growing according to older ages and to the presence of previous comorbidities, especially those that lead to some degree of cardiovascular impairment [2, 3].

As of 16 May 2020, the World Health Organization has reported 4 530 000 people infected with a total of 307 000 deaths worldwide. In Brazil, there are 220 000 confirmed cases and 14 962 deaths caused by the disease on the same date, which places the largest country in Latin America as the second in the world in terms of the rising epidemic curve of active cases and deaths [4].

The great challenge for Brazilian health authorities is to reduce the number of these cases to the minimum, especially the most serious ones, which require hospitalisation with the support of invasive mechanical ventilation. The international experiences of countries affected by the COVID-19 epidemic demonstrate that, once the installed capacity of health services is overcome, the disease can raise its lethality rate to worrying levels, possibly leading to the death of millions of people due to the total lack health care, especially in a scenario of economic crisis and intense social inequality, which makes millions of Brazilians living in underprivileged communities more susceptible to death [5].

In Brazil, hospital bed occupancy rates are already at 100% in some states and health professionals are beginning to have to adopt patient admission criteria based on their chances of survival. Complicated decisions as this one, which can mean life and death, need maximum scientific support in order to minimise the harm to all patients. Given this scenario, the current study aims to analyse the survival of inpatients and estimate the prognostic factors of patients admitted to Brazilian hospitals due to the COVID-19. Studies with this purpose, with a significant sample and with the level of scientific evidence of this design have not yet been published in the country.

Therefore, its findings may support the creation of scientifically supported clinical management protocols, minimising conflicting situations and subsidising decisions made in the hospital setting. In the face of a new disease that has no recognised treatment, vaccine or even its pathophysiology fully clarified, survival data and predictive factors are important instruments for the rational control of the epidemic and for scientific purposes, in order to better understand variables not yet discussed about COVID-19.

## Methods

### Study population and data collection

A retrospective, multicentre cohort study, of the survival analysis type, was carried out in order to estimate the prognostic factors of patients admitted to Brazilian hospitals due to COVID-19. Secondary, public and anonymous data were used from the notification forms of hospitalised patients and notified by SARS-COV-2 in hospitals in Brazil, in the observation period from 20 February 2020 to 2 June 2020. All hospitalised subjects and those confirmed with COVID-19 through the reverse transcription polymerase chain reaction (RT-PCR) exam were included in the study.

### Participants and variables

**Fig. 1.**
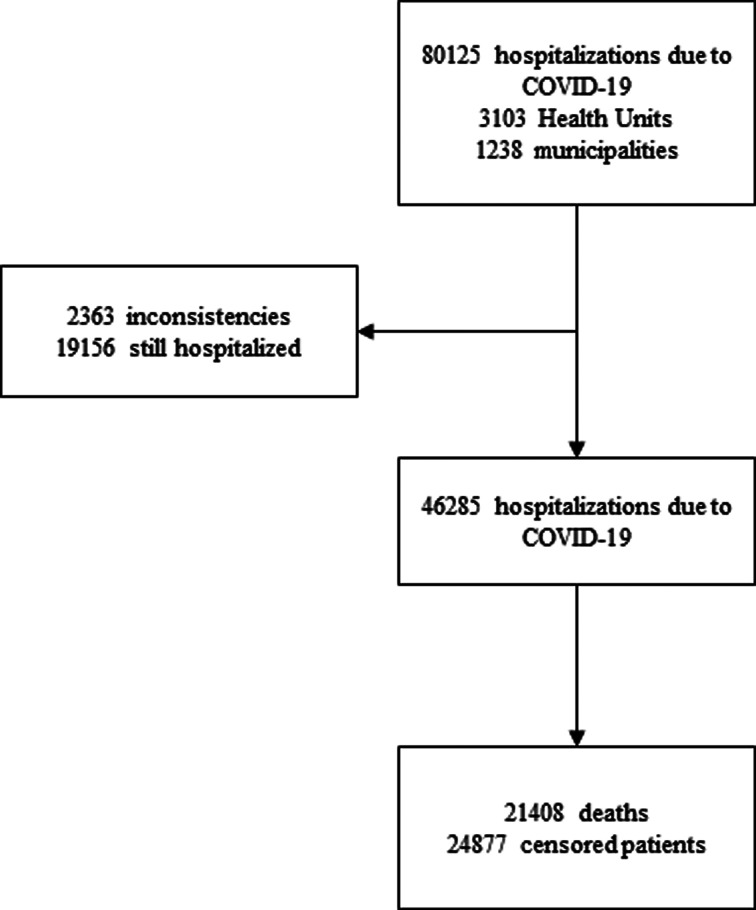
Flow diagram of the study.

There were 80 125 hospitalisations due to COVID-19 registered in the public national epidemiological surveillance system, obtained from the open access database [6], public and anonymous. The counting of the follow-up time starts from the first day of hospitalisation until the date of death or discharge. Records of subjects with missing hospitalisation dates or inconsistencies in their diagnostic record were excluded from the study. These represent 2.94% (2363/80 125) of the records. Censorship occurred in patients who were discharged or transferred, however, all records used in this study had an outcome definition until 2 June 2020, excluding from the cohort patients who were still hospitalised, representing 39.28% of the records (19 156/80 125) (Fig. 1).

The co-variables used to compare survival curves were socio-economic factors (age, sex, race, education and area of residence), clinical signs and symptoms (fever, cough, sore throat, diarrhoea and vomiting), hospital variables (influenza-like outbreak, hospital-acquired infection, dyspnoea, respiratory distress, O_2_ saturation <95%, intensive care unit (ICU) admission, ICU length of stay and X-ray result), chronic disease (heart disease, haematology, Down's syndrome, liver disease, asthma, diabetes mellitus, neurological disease, pneumopathy, immunodepression, kidney disease and body mass index (BMI)), if the patient has had a flu vaccine, use of antiviral against influenza and what is the type of such antiviral. The other variables on the notification form, whose completion was irregular on less than 5% of the cases, were excluded from the analysis (Supplementary form).

### Statistical analysis

The data were analysed from the estimates of survival functions, using the non-parametric Kaplan–Meier method [7]. The log-rank test and the Wilcoxon test were used to compare the survival functions for each socio-demographic covariate, signs or symptoms, hospital and clinical covariate. The numerical variable age was categorised by tertile and the categorical variables race and education were recategorised according to the similarity of the survival curves of each category, aggregating them by theoretical category. Due to the sample size, a value of *P* ⩽ 0.01 was considered to accept the significant differences in the survival curves.

To assess the risk factors associated with death, hazard ratios (HRs) and 95% confidence intervals (CIs) were calculated, following Cox's proportional hazards model [8]. Covariates with a *P*-value <0.250 were used to create the multiple models. The modelling was initiated by the most significant variables, both at the level of statistical significance and at the theoretical level, respecting the risk proportionality test and absence of multicollinearity to be able to test the variable in the model. The final model was tested, the device and Cox–Snell residues were analysed. The data were analysed using the STATA 12.0 program and in all analyses the level of significance was considered equal to 5%.

## Results

### Survival functions

Between 20 February 2020 and 2 June 2020, 46 285 hospitalisations due to COVID-19 were followed retrospectively. The median survival time was of 12 days (95% CI 11.82–12.18). At the end of the follow-up, 21 408 deaths (46.25%) and 24 877 (53.75%) censored patients were recorded (Fig. 1).

**Fig. 2.**
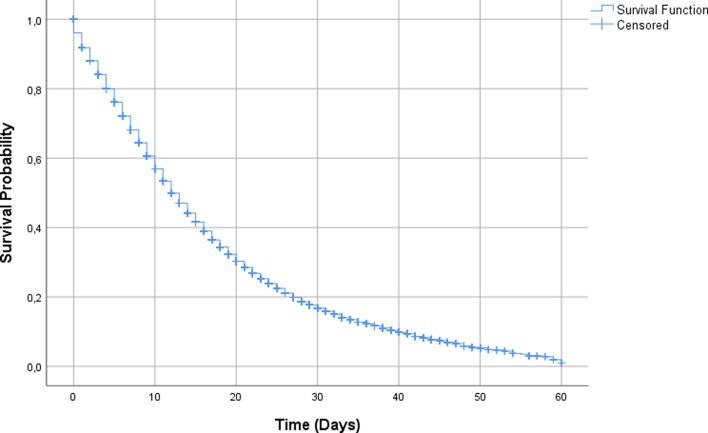
Overall survival of hospitalised patients.

The overall survival rate estimate was of 79.21% (95% CI 78.82–79.59) in 5 days of hospitalisation and 59.22% (95% CI 58.69–59.76) in 10 days (Fig. 2). In Table 1, when comparing the survival curves from the socio-demographic covariates, there is a significantly lower estimate of survival in 10 days in cases of ages above 68 years of 45.4% (95% CI 44.6–46.2), black or mixed race of 51.3% (95% CI 50.3–52.2), living in the countryside 48.8% (95% CI 45.0–52.5) and illiterate 37.4% (95% CI 34.0–40.9).

**Table 1.** Comparison of survival estimates for patients hospitalised due to SARS-CoV-2 at 5 and 10 days

	Deaths	%	5 days (%)	95% CI	10 days (%)	95% CI	Value of *P*
Age							
Up to 50 years old	3119	14.6	89.7	89.2–90.3	77.0	76.0–77.9	<0.001
51–67 years old	6763	31.6	81.1	80.5–81.8	62.5	61.5–63.4	
68 years old or more	11 526	53.8	69.2	68.5–69.9	45.4	44.6–46.2	
Sex							
Male	12 667	59.2	79.5	79.0–80.0	59.2	58.5–59.9	0.659
Female	8738	40.8	78.8	78.2–79.4	59.2	58.4–60.1	
Race							
White or Yellow	5930	42.9	83.8	83.2–84.4	65.8	64.9–66.7	<0.001
Black or mixed	7893	57.1	72.8	72.0–73.5	51.3	50.3–52.2	
Level of education							
Illiterate	633	10.0	62.9	59.6–66.0	37.4	34.0–40.9	<0.001
Low education	3201	50.7	74.4	73.2–75.5	53.7	52.3–55.2	
Intermediate/elementary education	1775	28.1	85.4	84.3–86.4	68.0	66.4–69.6	
Higher education	704	11.2	90.9	89.6–91.9	77.7	75.6–79.7	
Zone							
Urban	18 315	97.3	79.3	78.8–79.7	59.5	58.9–60.0	<0.001
Rural	503	2.7	70.2	67.1–73.2	48.8	45.0–52.5	
Influenza-like outbreak							
Yes	5961	39.7	76.8	76.0–77.6	56.4	55.4–57.5	<0.001
No	9047	60.3	80.8	80.2–81.3	61.9	61.1–62.7	
Hospital-acquired infection							
Yes	815	5.6	77.5	75.0–79.8	56.9	53.9–59.8	0.039
No	13 714	94.4	80.2	79.8–80.7	60.7	60.1–61.4	
Fever							
Yes	15 199	80.1	80.9	80.5–81.4	61.8	61.2–62.5	<0.001
No	3781	19.9	75.7	74.6–76.7	53.9	52.5–55.2	
Cough							
Yes	15 691	82.7	80.9	80.5–81.3	61.6	61.0–62.2	<0.001
No	3283	17.3	74.4	73.2–75.5	53.7	52.3–55.2	
Throat							
Yes	3768	25.4	82.0	81.2–82.8	63.3	62.1–64.5	<0.001
No	11 088	74.6	79.7	79.1–80.2	60.3	59.6–61.0	
Dyspnoea (shortness of breath)							
Yes	16 847	87.1	77.4	76.9–77.9	57.0	56.4–57.6	<0.001
No	2503	12.9	86.6	85.8–87.4	69.7	68.4–71.0	
Respiratory distress							
Yes	14 495	80.2	76.4	75.9–76.9	55.7	55.0–56.4	<0.001
No	3577	19.8	85.9	85.2–86.6	68.5	67.3–69.6	
O_2_ <95% saturation							
Yes	15 209	83.2	75.3	74.8–75.8	54.4	53.7–55.0	<0.001
No	3063	16.8	88.5	87.8–89.1	72.6	71.5–73.7	
Diarrhoea							
Yes	2406	16.6	83.5	82.5–84.4	64.9	63.4–66.3	<0.001
No	12 106	83.4	79.6	79.1–80.1	60.1	59.4–60.8	
Vomit							
Yes	1440	10.2	81.3	80.0–82.6	63.5	61.6–65.4	0.006
No	12 728	89.8	80.1	79.6–80.6	60.5	59.8–61.2	
Cardiopathy							
Yes	9032	70.9	76.4	75.7–77.1	54.4	53.5–55.2	<0.001
No	3705	29.1	78.8	77.9–79.7	60.2	58.9–61.5	
Haematological							
Yes	290	3.4	76.3	72.1–79.9	55.4	50.4–60.2	0.619
No	8335	96.6	77.4	76.7–78.0	56.8	56.0–57.7	
Down's syndrome							
Yes	73	0.9	70.9	61.3–78.5	38.5	28.0–48.8	<0.001
No	8512	99.1	77.2	76.5–77.9	56.7	55.8–57.5	
Hepatic							
Yes	298	3.5	72.5	68.2–76.4	51.1	46.0–55.9	0.036
No	8262	96.5	77.5	76.8–78.2	57.0	56.1–57.9	
Asthma							
Yes	488	5.7	85.6	83.5–87.4	69.5	66.3–72.4	<0.001
No	8149	94.3	76.9	76.2–77.5	56.2	55.3–57.0	
Diabetes							
Yes	6899	59.1	75.3	74.4–76.0	52.8	51.8–53.8	<0.001
No	4772	40.9	78.7	77.9–79.6	59.2	58.1–60.3	
Neurological							
Yes	1409	15.6	70.5	68.4–72.5	46.4	44.1–48.8	<0.001
No	7643	84.4	77.9	77.2–78.5	57.8	56.8–58.7	
Pneumopathy							
Yes	1266	14.1	75.4	73.4–77.3	52.2	49.8–54.6	<0.001
No	7694	85.9	77.6	76.9–78.3	57.4	56.5–58.3	
Immunodepression							
Yes	865	9.9	77.8	75.6–79.9	59.7	56.9–62.4	0.056
No	7883	90.1	77.2	76.5–77.9	56.7	55.8–57.6	
Kidney disease							
Yes	1556	17.2	73.1	71.1–74.9	48.4	46.2–50.6	<0.001
No	7512	82.8	77.6	76.9–78.3	57.6	56.7–58.5	
BMI							
Normal	7	0.9	91.7	53.9–98.8	46.9	17.6–71.8	0.117
Overweight	52	6.7	81.5	71.8–88.1	50.0	37.8–61.1	
Obesity I	373	47.9	82.0	79.1–84.7	62.6	58.6–66.3	
Obesity II	192	24.6	82.1	77.7–85.7	60.0	54.1–65.5	
Obesity III	155	19.9	76.7	71.2–81.2	52.9	46.0–59.4	
Vaccine							
Yes	2371	32.1	80.9	79.8–82.0	62.2	60.7–63.8	0.521
No	5012	67.9	81.1	80.3–81.8	62.4	61.4–63.5	
Anti-viral							
Yes	8324	51.3	81.3	80.7–81.8	61.1	60.3–62.0	0.068
No	7889	48.7	80.1	79.5–80.7	61.3	60.4–62.1	
Antiviral type							
Oseltamivir	7675	97.3	81.5	80.9–82.1	61.4	60.6–62.3	0.591
Zanamivir	35	0.4	76.2	64.0–84.7	50.0	35.4–63.0	
Other	178	2.3	82.7	78.5–86.1	64.5	58.7–69.8	
ICU							
Yes	11 070	58.5	77.1	76.5–77.8	54.5	53.7–55.3	<0.001
No	7869	41.5	82.5	82.0–83.0	66.2	65.4–66.9	
Ventilatory support							
Yes, invasive	2439	13.4	66.1	65.2–67.0	41.6	40.7–42.6	<0.001
Yes, not invasive	6525	35.9	82.7	82.1–83.3	65.4	64.6–66.3	
No	9202	50.7	90.3	89.7–90.8	77.6	76.5–78.6	
X-ray							
Normal	406	6.0	87.1	85.0–88.9	71.5	68.3–74.5	<0.001
Infiltrate interstitial	4732	70.2	80.3	79.5–81.1	60.9	59.8–62.0	
Consolidated	735	10.9	80.5	78.3–82.5	60.5	57.6–63.3	
Mixed	866	12.9	79.8	77.8–81.7	60.5	57.8–63.0	
ICU length of stay							
Up to 8 days	6418	58.9	57.0	55.9–58.0	15.0	14.0–16.0	<0.001
>8 days	4486	41.1	95.2	94.7–95.6	89.5	88.8–90.2	

Comparing at the same cutoff point in 10 days (Table 1), survival estimates were significantly lower when the subjects had dyspnoea 57.0% (95% CI 56.4–57.6), respiratory distress 55.7% (95% CI 55.0–56.4), O_2_ saturation <95% 54.4% (95% CI 53.7–55.0), mixed X-ray alteration 60.5% (95% CI 57.8–63.0), were admitted to the ICU 54.5% (95% CI 53.7–55.3), when the length of stay in the ICU was up to 8 days 15.0% (95% CI 14.0–16.0), when they had invasive ventilatory support 41.6% (95% CI 40.7–42.6) and influenza-like outbreak 56.4% (95% CI 55.4–57.5). Still in Table 1, there was a lower estimate of the 10-day survival rate when the patient had chronic disease, such as heart disease 54.4% (95% CI 53.5–55.2), neurological 46.4% (95% CI 44.1–48.8), pneumopathy 52.2% (95% CI 49.8–54.6), diabetes 52.8% (95% CI 51.8–53.8) and kidney disease 48.4% (95% CI 46.2–50.6) and Down's syndrome 38.5% (95% CI 28.0–48.8).

The covariates of clinical signs showed a better estimate of the survival of patients in 10 days when they had fever 61.8% (95% CI 61.2–62.5), cough 61.6% (95% CI 61.0–62.2), sore throat 63.3% (95% CI 62.1–64.5), diarrhoea 64.9% (95% CI 63.4–66.3) or vomiting 63.5% (95% CI 61.6–65.4). In the same comparison, patients who reported having asthma had a better survival estimate of 69.5% (95% CI 66.3–72.4).

### Cox's proportional

In Table 2, after assessing the proportional risks, the statistically significant covariates that presented the highest risk of death were the age of 68 years HR 2.77 (95% CI 2.66–2.88), black or mixed race HR 1.54 (95% CI 1.49–1.59), living in the countryside HR 1.26 (95% CI 1.16–1.38), illiterate HR 3.29 (95% CI 2.95–3.66), dyspnoea HR 1.46 (95% CI 1.40–1.53), respiratory distress HR 1.48 (95% CI 1.43–1.54), O_2_ saturation <95% HR 1.74 (95% CI 1.67–1.81), mixed X-ray alteration HR 1.42 (95% CI 1.26–1.59), admitted to the ICU HR 1.38 (95% CI 1.34–1.43), length of stay in the ICU up to 8 days HR 9.15 (95% CI 8.73–9.60), invasive ventilatory support HR 2.94 (95% CI 2.81–3.07), influenza-like outbreak HR 1.18 (95% CI 1.14–1.22), heart disease HR 1.18 (95% CI 1.13–1.22), neurological HR 1.31 (95% CI 1.24–1.39), pneumopathy HR 1.11 (95% CI 1.05–1.18), diabetes HR 1.19 (95% CI 1.14–1.23), kidney disease HR 1.25 (95% CI 1.19–1.32) and Down's syndrome HR 1.51 (95% CI 1.20–1.90).

**Table 2.** Comparison of Cox proportional HR in relation to the risk of death

	*n*	HR	95% CI	*P*
Age				
Up to 50 years old	27 932	1.00		
51–67 years old	25 747	1.74	1.67–1.82	<0.001
68 years old or more	26 444	2.77	2.66–2.88	<0.001
Sex				
Male	45 926	1.01	0.98–1.03	0.668
Female	34 176	1.00		
Race				
White or Yellow	24 091	1.00		
Black or mixed	26 614	1.54	1.49–1.59	<0.001
Level of education				
Higher education	5223	1.00		
Intermediate/elementary education	8954	1.49	1.36–1.62	<0.001
Low education	10 391	2.23	2.06–2.42	<0.001
Illiterate	1630	3.29	2.95–3.66	<0.001
Zone				
Urban	68 418	1.00		
Rural	1864	1.26	1.16–1.38	<0.001
Influenza-like outbreak				
Yes	19 609	1.18	1.14–1.22	<0.001
No	35 911	1.00		
Hospital-acquired infection				
Yes	2143	1.07	1.00–1.15	0.044
No	52 180	1.00		
Fever				
Yes	58 977	0.79	0.77–0.83	<0.001
No	13 132	1.00		
Cough				
Yes	61 368	0.79	0.76–0.82	<0.001
No	10 817	1.00		
Throat				
Yes	17 084	0.88	0.85–0.91	<0.001
No	40 740	1.00		
Dyspnoea (shortness of breath)				
Yes	55 650	1.46	1.40–1.53	<0.001
No	15 218	1.00		
Respiratory distress				
Yes	46 460	1.48	1.43–1.54	<0.001
No	19 326	1.00		
O_2_ <95% saturation				
Yes	44 351	1.74	1.67–1.81	<0.001
No	21 198	1.00		
Diarrhoea				
Yes	10 881	0.86	0.82–0.90	<0.001
No	45 191	1.00		
Vomit				
Yes	6128	0.93	0.88–0.98	0.007
No	48 436	1.00		
Cardiopathy				
Yes	25 160	1.18	1.13–1.22	<0.001
No	12 619	1.00		
Haematological				
Yes	759	1.03	0.91–1.16	0.629
No	25 487	1.00		
Down's syndrome				
Yes	200	1.51	1.20–1.90	<0.001
No	25 911	1.00		
Hepatic				
Yes	680	1.13	1.01–1.26	0.042
No	25 372	1.00		
Asthma				
Yes	2238	0.66	0.61–0.73	<0.001
No	24 492	1.00		
Diabetes				
Yes	18 872	1.19	1.14–1.23	<0.001
No	15 655	1.00		
Neurological				
Yes	3007	1.31	1.24–1.39	<0.001
No	24 212	1.00		
Pneumopathy				
Yes	2935	1.11	1.05–1.18	<0.001
No	24 148	1.00		
Immunodepression				
Yes	2405	0.93	0.87–1.00	0.063
No	24 237	1.00		
Kidney disease				
Yes	3258	1.25	1.19–1.32	<0.001
No	23 729	1.00		
BMI				
Normal	17	1.00		
Overweight	139	1.18	0.53–2.61	0.675
Obesity I	1227	1.00	0.47–2.12	0.990
Obesity II	616	1.16	0.54–2.45	0.706
Obesity III	475	1.27	0.59–2.70	0.539
Vaccine				
Yes	9914	1.01	0.97–1.07	0.532
No	21 723	1.00		
Anti-viral				
Yes	26 461	0.97	0.94–1.00	0.076
No	34 881	1.00		
Antiviral type				
Oseltamivir	23 906	1.00		
Zanamivir	114	1.13	0.81–1.57	0.473
Other	910	0.95	0.82–1.10	0.491
ICU				
Yes	24 284	1.38	1.34–1.43	<0.001
No	40 834	1.00		
Ventilatory support				
Yes, invasive	23 638	2.94	2.81–3.07	<0.001
Yes, not invasive	27 900	1.59	1.52–1.66	<0.001
No	14 806	1.00		
X-ray				
Normal	2173	1.00		
Infiltrate interstitial	16 003	1.42	1.28–1.57	<0.001
Consolidated	2277	1.42	1.26–1.61	<0.001
Mixed	2723	1.42	1.26–1.59	<0.001
ICU length of stay				
Up to 8 days	9452	9.15	8.73–9.60	<0.001
>8 days	7588	1.00		

Patients had a better prognosis when they had signs or symptoms such as fever HR 0.79 (95% CI 0.77–0.83), cough HR 0.79 (95% CI 0.76–0.82), sore throat HR 0.88 (95% CI 0.85–0.91), diarrhoea HR 0.86 (95% CI 0.82–0.90), vomiting HR 0.93 (95% CI 0.88–0.98) or asthma HR 0.66 (95% CI 0.61–0.73) (Table 2). No significant difference was found in the survival curves of the sex co-variables, hospital-acquired infection, if the patient had previous haematological disease, liver disease, immunodepression, BMI, if they had been vaccinated against influenza, use antiviral or some type of antiviral.

**Fig. 3.**
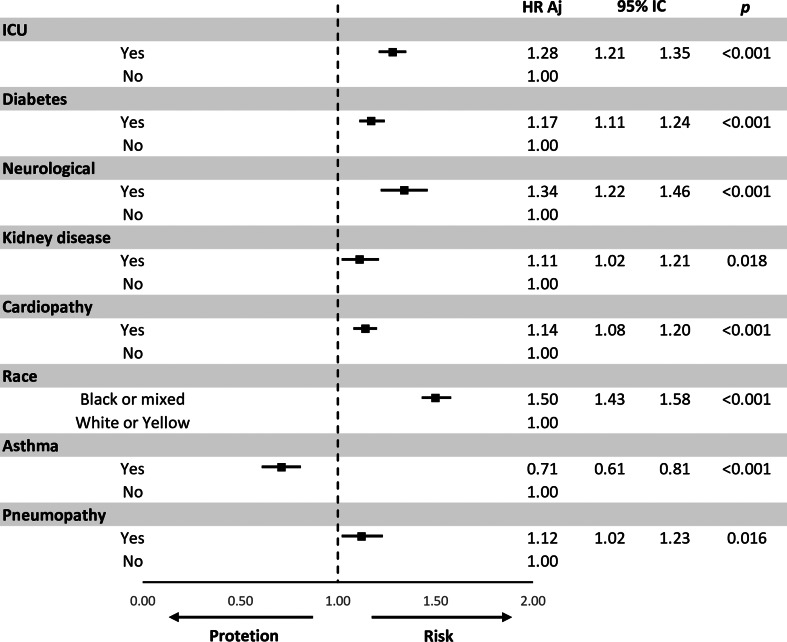
Multivariate model for the comparison of risks (HR) adjusted for death.

After adjusting for confounding variables, they remained significantly in the multiple model for increased risk of death when they were admitted to the ICU HR 1.28 (95% CI 1.21–1.35), diabetes HR 1.17 (95% CI 1.11–1.24), neurological disease HR 1.34 (95% CI 1.22–1.46), kidney disease HR 1.11 (95% CI 1.02–1.21), heart disease HR 1.14 (95% CI 1.08–1.20), black or mixed race of HR 1.50 (95% CI 1.43–1.58), asthma HR 0.71 (95% CI 0.61–0.81) and pneumopathy HR 1.12 (95% CI 1.02–1.23) (Fig. 3).

## Discussion

This multicentre retrospective cohort study of patients hospitalised with COVID-19 found important differences in survival times, as well as risk factors or protection for the death of patients in Brazilian hospitals. There was a reduced overall survival time, in addition to an association with lower survival estimates and a higher risk of death related to age, race, education, zone, dyspnoea, respiratory distress, influenza-like outbreak, O_2_ saturation, X-ray changes of chest, ICU stay, length of stay in ICU, use of ventilatory support, presence of heart disease, diabetes, Down's syndrome, pneumopathy, kidney disease and neurological disease. The variables of clinical signs or symptoms, such as fever, cough, sore throat, diarrhoea or vomiting, showed greater survival and less risk of death. Finally, the presence of asthma had a better prognosis.

The overall survival of patients hospitalised for treatment of COVID-19 in Brazil was of 62.05%. This survival is shown to be lower than other survival studies of hospitalised patients in Brazil. A retrospective cohort study developed by Bonfada *et al*., whose objective was to analyse the survival of elderly patients admitted to the ICU in a low-development region, found an overall survival of 66.64%, which reinforces the concern about the lethality of COVID-19 in countries with limited resources [9].

The lack of care and clinical management protocols for patients hospitalised by COVID-19 in Brazil, closely linked to internal political disputes in the country, and the consequent delay in training the health teams have a direct influence on this context. In addition, there is no strategy for testing the asymptomatic population and even for those with suggestive symptoms there is a delay in the diagnosis of about 10 days for the confirmatory result via RT-PCR, a time interval in which there is already a drop in survival to 62.05%.

In our study when comparing survivals by socio-demographic covariates, a worse prognosis was found for patients over 68 years of age, with 2.77 times the risk of death. Besides, it was found that black or mixed-race people had a risk of death 1.54 times bigger, and illiterate people 3.29 times. All consecutive patients diagnosed with COVID-19 admitted to the Renmin Hospital of the Wuhan University, were enrolled in a retrospective cohort study. One-third of the patients got better in hospital during follow-up. Twenty-five patients died, the mortality rate was 3.77%. In this study, older patients were prone to have severe COVID-19 symptoms and worsen, and were more likely to die in the hospital [10]. Another cohort study identified several risk factors for death in adults who were hospitalised with COVID-19. Older age was also associated with higher odds of in-hospital death, it revealed age ⩾65 years as a strong predictor for death from COVID-19 pneumonia [11, 12].

According to the Brazilian Institute of Geography and Statistics (IBGE), Brazil has a population of about 211 million inhabitants, among which there are 25 million elderly people and 55 million people who are below the poverty line, a group that is mostly composed by black people with low education [13]. Populations that live under vulnerability or social inequity often have less access to information and health services and they may reach hospital services late. In the same way, most of the people working as self-employed or underemployed, are forced out of social isolation, often without protection, to ensure the livelihood of their families, what makes them subject to potential infection rates even higher.

In this sense, health policies need to have access to the information considered above to promote an adequate confrontation with reality, especially by creating protection mechanisms for these vulnerable groups. Otherwise, the COVID-19 epidemic in Brazil runs the risk of being a catalyst that assumes a higher risk of death of 26% to its entire population, indirectly characterised as a means of eugenics, based on socio-economic and demographic criteria, worrying and incongruent with the constitutional principles and the guarantee of the principle of equity in health care, one of the pillars of the Brazilian public health system. Underprivileged people are at higher risk of infection and death from COVID-19, and they have less access to care due to systems that treat health as a commodity and not a human right [14, 15].

When assessing the context of clinical variables, significance was found for better survival when patients presented signs or symptoms at the time of hospital admission, such as fever, cough, vomiting, sore throat or diarrhoea, showing they may still be in the viral replication phase, therefore, the presence of signs or symptoms in this phase could be less severe. In addition, it is likely that patients will have a less severe reaction by the immune system, which would differ from other patients who may progress abruptly to clinical severity, without having any of these symptoms at the beginning of the hospitalisation.

COVID-19 pneumonia is a multistate disease with clinically relevant intermediate endpoints like severity onset. Most survival data analyses set this onset as the primary endpoint, and censor recovery or hospital discharge. However, when competing risks of severity onset are present, this analytical method induces bias. The risk of severe progression assessed without considering the competition would be overestimated because the patients who would never progress (those who discharged from the hospital without progression) were treated as if they could progress [16].

It has been shown that the ICU length of stay of up to three days has the worst prognosis, which reinforces our hypothesis that patients worsen their clinical condition abruptly during hospitalisation, and admission to the ICU would have little therapeutic capacity in more severe cases of COVID-19. However, it would be necessary to develop prospective cohort studies to monitor, mainly, the changes in the clinical signs of patients with COVID-19 and their impact on the survival of hospitalised patients and how this interferes with the length of stay in the ICU.

The level and duration of infectious virus replication are important factors in assessing the risk of transmission and guiding decisions regarding isolation of patients. Because the coronavirus RNA detection is more sensitive than the virus isolation, most studies have used qualitative or quantitative viral RNA tests as a potential marker for infectious coronavirus. The detectable SARS-CoV-2 RNA persisted for a median of 20 days in survivors and it was sustained until death in non-survivors [11].

In addition to clinical symptoms, an important result found in this study was asthma as a protective factor against death. Interestingly, the prevalence of asthma in patients with COVID-19 was of 0.9% in an ambispective cohort study of consecutive hospitalised patient. Thus, it was speculated that TH2 immune response in patients with asthma may counter the inflammation process induced by the SARS-CoV-2 infection. Further studies are required to characterise the immune response and inflammation features of COVID-19 [17].

Hospitalised patients in the No. 7 Hospital of Wuhan, clinically diagnosed with ‘viral pneumonia’ based on their clinical symptoms (fever or respiratory symptoms) with typical changes in chest radiology, were preliminarily involved in a study. No asthmatic patient was identified in this report, and only a few patients had self-reported drug hypersensitivity and urticaria. Virus infections have been associated with acute exacerbation of asthma, and allergy may not be a risk factor for virus infection. It is plausible that this concept may also apply to SARS-CoV-2; however, having no asthma patients and no respiratory allergies does not support this concept at least in this series of 140 patients [18].

Regardless of the socio-demographic variables, signs and symptoms, when analysing the risks in the multivariate model, patients who were admitted to the ICU or who had diabetes or heart, kidney and neurological diseases as comorbidities had the worst prognosis.

Most severe patients with COVID-19 admitted to the Tongji Hospital that were retrospectively enrolled and followed-up showed rapid progression and multiple organ dysfunction. During hospitalisation, a substantial proportion of patients presented cardiac injury, liver and kidney dysfunction and hyperglycaemia. This current study also revealed that hyperglycaemia was related to increased mortality in patients with COVID-19. The prevalence of hyperglycaemia may be associated with underlying diabetes and corticosteroid therapy [17].

Led by the National Health Commission of the People's Republic of China, a retrospective cohort was established, to study COVID-19 admitted cases from 575 hospitals throughout China [19]. Non-survivors present a higher proportion of various coexisting chronic illnesses in the univariate analysis. Coronary heart disease and cerebrovascular disease are confirmed to be independent risk factors for death. Another study in Wuhan Pulmonary Hospital revealed that underlying cardiovascular or cerebrovascular diseases contributed to high mortality from COVID-19 pneumonia [12].

There were 220 patients with confirmed COVID-19 recruited from the Union Hospital of Huazhong University of Science and Technology. The current study showed that 161 COVID-19 patients (80.5%) presented at least one of the comorbidities, including diabetes, hypertension, hepatic disease, cardiac disease, chronic pulmonary disease and others. Interestingly, COVID-19 patients with chronic pulmonary disease, mainly chronic obstructive pulmonary disease, obviously presented elevated risk of death. It was found that there was a trend for hypertension and diabetes to elevate the risk of death from COVID-19 pneumonia [20].

In this aspect, it is necessary to develop strategies that aim to make a consistent clinical assessment on the organic impacts in patients with SARS-CoV-2 infection and comorbidities. Because of these, of course, there is already a higher risk of death on hospitalisation due to factors such as a greater inflammatory response, propensity to chronic endothelial lesions and intravascular coagulation, acid/basic blood imbalance and impaired immune response.

Although the study with secondary data notification forms has its limitations, this problem is partially solved, mainly due to the urgency of the country in maintaining more accurate data in the monitoring of COVID-19, so that health institutions are able to plan actions to control the epidemic. In some cases, there was incomplete documentation of the history and symptoms in the electronic database, even after making efforts regarding feedback and recollection. Some diagnoses of co-existing illnesses were originated in self-reports from patients at their admission, which might lead to recall bias.

However, this study has a robust methodology, with appropriate data analysis and the first research in the country with all hospitalisation in this gap of time. Furthermore, this research confirmed the importance of age and the presence of chronic diseases not only in the incidence of more serious cases of COVID-19, but also its effects on the drop in the survival curve of patients in hospitals in Brazil, which is considered one of the worldwide epicentres to COVID-19 nowadays.

In conclusion, it is considered to be important that scientifically supported protocols are developed for the management of appropriate clinical care for patients in hospital due to the COVID-19, especially in the face of a scenario where there is no consensus on pharmacological treatment or even therapeutic management. It is worth mentioning the importance in the development of new studies, such as controlled and randomised clinical trials, in order to reaffirm the results of the current study, especially regarding the variable asthma as a protective factor against death.

## Data Availability

The data that support the findings of this study will be available on request and permission of via e-mail from the corresponding author.
